# Support Vector Data Description Model to Map Specific Land Cover with Optimal Parameters Determined from a Window-Based Validation Set

**DOI:** 10.3390/s17050960

**Published:** 2017-04-26

**Authors:** Jinshui Zhang, Zhoumiqi Yuan, Guanyuan Shuai, Yaozhong Pan, Xiufang Zhu

**Affiliations:** 1 Department of Geography, Beijing Normal University, State Key Laboratory of Earth Surface Processes and Resource Ecology, Beijing 100875, China; zhangjs@bnu.edu.cn (J.Z.); pyz@bnu.edu.cn (Y.P.); zhuxiufang@bnu.edu.cn (X.Z.); 2 Department of Resources Science and Technology, Beijing Normal University, College of Resources Science and Technology, Beijing 100875, China; 3 Department of Earth and Environmental Sciences, Michigan State University, East Lansing, MI 48824, USA; sgy@mail.bnu.edu.cn

**Keywords:** support vector data description, optimal parameters, window-based validation set, simulated annealing, land cover

## Abstract

This paper developed an approach, the window-based validation set for support vector data description (WVS-SVDD), to determine optimal parameters for support vector data description (SVDD) model to map specific land cover by integrating training and window-based validation sets. Compared to the conventional approach where the validation set included target and outlier pixels selected visually and randomly, the validation set derived from WVS-SVDD constructed a tightened hypersphere because of the compact constraint by the outlier pixels which were located neighboring to the target class in the spectral feature space. The overall accuracies for wheat and bare land achieved were as high as 89.25% and 83.65%, respectively. However, target class was underestimated because the validation set covers only a small fraction of the heterogeneous spectra of the target class. The different window sizes were then tested to acquire more wheat pixels for validation set. The results showed that classification accuracy increased with the increasing window size and the overall accuracies were higher than 88% at all window size scales. Moreover, WVS-SVDD showed much less sensitivity to the untrained classes than the multi-class support vector machine (SVM) method. Therefore, the developed method showed its merits using the optimal parameters, tradeoff coefficient (*C*) and kernel width (*s*), in mapping homogeneous specific land cover.

## 1. Introduction

Land-cover thematic maps produced with remote sensing images have been used to record the Earth’s surface development processes and dramatic land use/cover changes impelled from natural and human factors [1,2,3]. Typically, exhaustively-labeled training data within images are required to map all land-cover types by supervised classification methods. Omission of any class would degrade classification performance because a pixel belonging to an untrained class would be erroneously allocated to one of the pre-defined classes in the training set [4]. For many applications, however, users are concerned about one specific class, such as the wetland or urban class. In such cases, conventional multi-class classification makes more of an effort to select training samples of non-target classes to meet the requirement of an exhaustive training set [5,6]. Much of the research has converted the solution from the traditional multi-class classification model to a one-class classification model which defines the user-interested class as a target, and other classes as outliers. The one-class classifier is designed to extract the interested land-cover class using a small training set including only the target class, thus it can efficiently reduce the hard and redundant work to collect all classes of ground training data for multi-class classification [7,8,9,10].

The support vector data description (SVDD) method, a boundary method developed by Tax and Duin, creates a hypersphere which is the decision boundary in a high-dimensional feature space such that it encloses most target objects and rejects outliers [11]. This method shows excellent ability in mapping specific land-cover distribution [12,13,14]. Foody and Mathur [15] demonstrated that the SVDD could achieve satisfying land-cover accuracy, which had little difference in terms of accuracy compared with multi-class support vector machine (SVM) classifications. Sanchez-Hernandez et al. [16] introduced SVDD classification to map fenland, which outperformed the conventional multiclass maximum-likelihood classification algorithm. Muñoz-Marí et al. [17] also analyzed and compared the applicability of different one-classifiers, and concluded that the SVDD classifier yielded the best crop classification with respect to other one-classifiers when applied to multi-spectral, hyperspectral and SAR (Synthetic Aperture Radar) data. Niazmardi et al. [13] combined Fuzzy C-means with SVDD for unsupervised hyperspectral data classification, which obtained acceptable results with high dimensional data. Uslu et al. [18] presented ensemble methods for improving classification performance of SVDD in the remotely sensed hyperspectral imagery data.

Based on the SVDD’s principle, the tradeoff coefficient *C* and kernel width *s*, are two critical parameters that affect the shape of hypersphere [19]. The *C* is defined as the ratio of target objects to outlier objects in a training sample set and the kernel width *s* is to control the compactness of hypersphere. When the s value is fixed, the reduction of *C* causes a shrinking hypersphere and more target objects would be rejected as outliers. When *C* is set as constant, smaller s contributes to an over-tight boundary around the training sample set whereas a very loose one would be derived with higher *s* value. Previous research demonstrated that the accuracy of one-class classification is very sensitive to these parameters. In particular, when the spectral mixture between the target class and outliers is significant, classification errors may be relatively high with inappropriate parameters. However, little attention was drawn to determining the appropriate parameters, *C* and *s*, for the SVDD model to ensure land-cover mapping performance.

In this paper, an innovative approach was developed to improve SVDD performance for specific land cover by optimizing classification parameters using a window-based validation set. The simulated annealing (SA) search algorithm was employed to determine optimal parameters. Hereafter, the method of window-based validation set for SVDD is abbreviated as WVS-SVDD. The remainder of this paper is organized as follows. The modules of the proposed method are introduced in Section 2. Then, experiments are conducted to test the performance of WVS-SVDD on different spatial resolution remote sensing images in Section 3. Finally, the conclusions are drawn in Section 4.

## 2. WVS-SVDD: Window-Based Validation Set for SVDD

**Figure 1:**
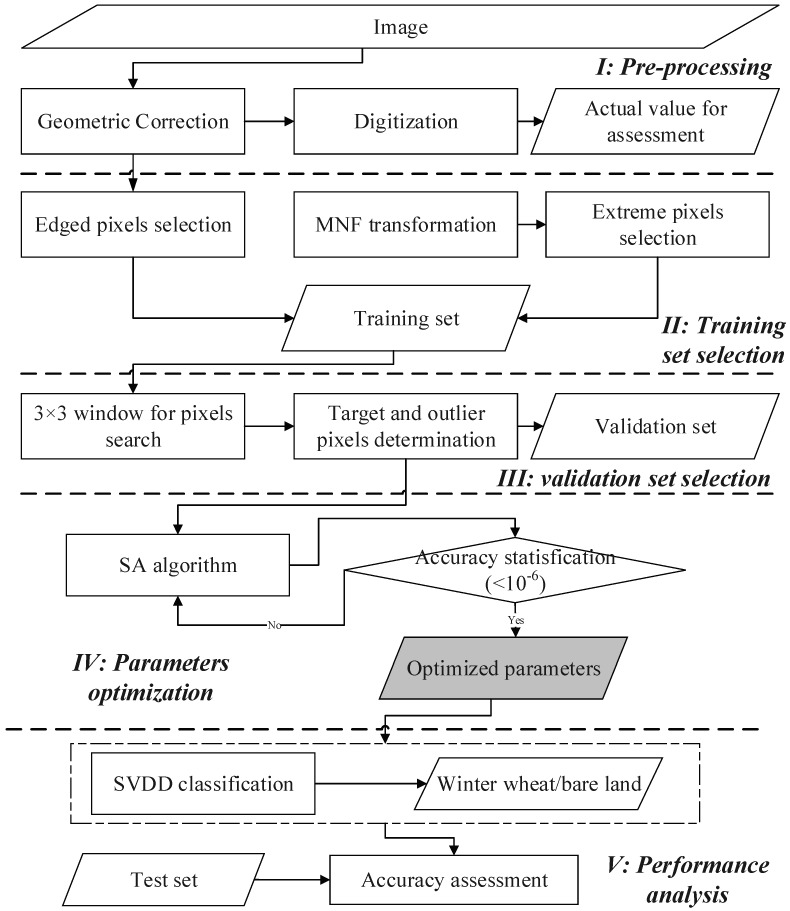
Flow chart of support vector data description (SVDD) for land cover mapping using the optimal parameters. The input and regenerated data delineated by rhomboid. MNF: minimum noise fraction; SA: simulated annealing.

The proposed method includes four modules (Figure 1): (1) training set selection; (2) validation set selection; (3) optimized parameter determination using simulated annealing (SA) algorithm; and (4) SVDD-based specific land-cover classification. Each module is described in the following sections.

### 2.1. Training Set Selection

The size and spectral feature of training set are of great importance for supervised classification to ensure the land-cover thematic map accuracy. The training set size is positively related to the classification accuracy [20]. Following the rule of thumb, a training set comprising at least 30*p* pixels (*p* is the number of the wavebands of remote sensing image) is considered enough to represent the spectral characteristics of the target class [21,22]. The spectral feature of training set is also important since the hypersphere is fitted around the training set. Edge spectral responses from the target land-cover spectral space are demonstrated to provide potential and effective support vectors, which are more effective than training samples that lie in the center of spectral cluster of the target class [23]. From our previous study, the edge training set consisted of two parts of sources: mixed and corner pixels. The mixed pixels are situated around target land-cover parcel boundaries where pixels represent the mixed spectra of the target class and other classes [24]. The corner pixel set are defined as the pixels lying at the vertex of the convex data from the view of spectral feature space constructed by the first and second component generated from the minimum noise fraction (MNF) transformation scatterplots [25]. These pixels together are potential support vectors for creating an optimal hypersphere.

### 2.2. Validation Set Selection

Cross validation is a common way to determine optimal parameters for multi-class classification. The training set itself is randomly split into different subsets in the first step. Then, some subsets are used to train the classifier with different parameter sets, which is validated by the remaining subsets. This method is not suitable for one-class classification because only pixels from target class are included in the training set. Any hypersphere large enough to enclose the whole training set would be considered as a suitable one, which may be easily to include outlier pixels when applied to the whole image. Therefore, rather than using training set, an independent validation set including both target class pixels and outlier pixels should be selected. The target pixels are used to examine whether the hypersphere could enclose the target class and outlier pixels could measure the ability of the hypersphere for rejecting other land-cover classes.

**Figure 2:**
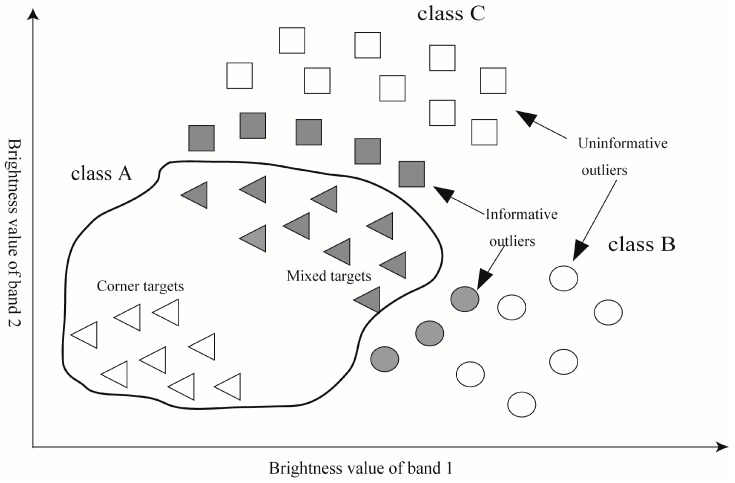
Definition of informative outliers from feature space. Circles and squares indicate cases of class of no interest (class B and class C) respectively, and triangles indicate cases of class of interest (class A). The solid line indicates the hypersphere fitted by target pixels. Solid circles and squares are located around the hypersphere, denoted as informative outliers.

According to Wang et al.’s research, the location of outlier objects in the validation set is crucial to construct the optimal hypersphere [26]. Figure 2 shows the distribution of the expected outliers from the view of the feature space, outlier pixels which are located tightly adjacent to the mixed target cases are needed. A method proposed by Wang et al. firstly utilized the boundary value method to generate artificial outliers which surrounded the target data set [26]. However, these artificial outliers were unable to represent actual scene and usually resulted in an overfitting hypersphere.

In this paper, a window-based sampling method was proposed to acquire neighboring pixels of training set as the validation set. Our method is based on the phenomenon that each mixed pixel straddling a boundary represents the two mixed spectral responses, which is dominated by each of the classes separated by that boundary [24]. The mixed spectral responses of two classes are also adjacent in the feature space, which could provide outliers neighboring to training samples to constrain the hypersphere, as illustrated in Figure 2. In the previous section, mixed target pixels are included in the edge training samples; their neighboring pixels thus provide informative outlier candidates. Accordingly, the window-based sampling method fits a window to each training pixel, the neighboring pixels of training samples within this window were collected to comprise validation set.

### 2.3. Optimal C and s Determination Using SA Algorithm

With training and validation sets, the optimal parameters can be determined by adaptive optimization algorithms. The SA as a global optimization algorithm was introduced here to avoid local optimality during each iteration [27]. Based on the Boltzmann and Metropolis’s study, Kirkpatrick et al. claimed that the Metropolis’s approach was conducted for each temperature on the annealing schedule until thermal equilibrium was reached. The “cooling” process, analogical to the cooling of a metal, enables SA to converge gradually to the search outcomes to accomplish global optimization. The SA algorithm has already been proposed for SVM parameters and feature selection, resulting in higher classification performance of SA-SVM than that of grid search which is also a popular parameters search method for SVM [28]. We introduced SA to determine optimal *C* and *s* for SVDD. The main steps are described as follows:

Step 1 (Initialization). The upper and lower bounds of *C* and *s* were set as (0.01, 1) and (0.01, 20), respectively. Within the parameter scope, the hypersphere could dilate from an underestimated state to an overestimated one, therefore enabling SA to search optimal parameters for SVDD. The SA algorithm starts generating and feeding initial values of the two parameters into the SVDD classifier, then, the classifier is applied to the test set and the system state (E_0_) is calculated.

In this research, the focus was on the target class, thus corrected classified outliers were not considered in the definition of the system state. The value of classification error defined from Formula (1) serves as the criterion to determine suitable parameters for the SVDD model.

(1) $$Error=\frac{\#wrong}{\#correct+\#wrong}$$

where #*correct* and #*wrong* denote the number of correctly classified target pixels and misclassified pixels, respectively.

Step 2 (Provisional state). Make a random move to change the existing system state to a provisional state. Another set of two positive parameters is generated in this stage.

Step 3 (Acceptance tests). The following equation was employed to determine the acceptance or rejection of the provisional state:

(2) $$\left\{ \begin{array}{l} \mathrm{Accept}\;\mathrm{the}\;\mathrm{provisional}\;\mathrm{state}\;\mathrm{if}\;E(s_{\mathrm{new}})>E(s_{\mathrm{old}}),\;\mathrm{and}\;p<P(\mathrm{accept}\;s_{\mathrm{new}}),\;0\leq p\leq 1\\ \mathrm{Accept}\;\mathrm{the}\;\mathrm{provisional}\;\mathrm{state}\;\mathrm{if}\;E(s_{\mathrm{new}})\leq E(s_{\mathrm{old}})\\ \mathrm{Reject}\;\mathrm{the}\;\mathrm{provisional}\;\mathrm{state}\;\mathrm{otherwise} \end{array}\right.$$

where $P(\mathrm{accept}\;s_{\mathrm{new}})$ is the Boltzmann probability factor, which equals e(−(E(snew)−E(sold))kbT) where *k_b_* is the Boltzmann constant and *T* is the current temperature. *p* is a random number to determine the acceptance of the provisional state, and *S*_new_ and *S*_old_ are new and original system state, respectively. If the provisional state is accepted, then set the provisional state as the current state.

Step 4 (incumbent solutions). If the provisional state is not accepted, then return to Step 2. Furthermore, if the current state is not superior to the system state, then repeat Steps 2 and 3 until the current state is superior to the system state and, finally, set the current state as the new system state.

Step 5 (temperature reduction). After the new system state is obtained, reduce the temperature. The new temperature reduction is obtained by:

(3) New temperature=(Current temperature)×ρ, where 0<ρ<1

where *ρ* is the cooling coefficient and is set as 0.9 in this study. If the pre-determined condition is reached, then the algorithm stops, and the latest state is an approximate optimal solution for each parameters. Otherwise, go to Step 2.

### 2.4. SVDD-Based Specific Land-Cover Classification

SVDD classification using the above training set and optimal parameters generated from SA model is then implemented to produce specific land cover map.

## 3. Experiments

In this section, we analyzed the strengths and weaknesses of the proposed WVS-SVDD method. In the first part of experiments, the comparison between WVS-SVDD, conventional SVDD and binary SVM classification was made to test the performance of the proposed method for mapping wheat and bare land maps, respectively. Then, we tested the robustness of the WVS-SVDD using different-size validation sets on multi-scale images. In addition, Foody et al. verified that the presence of untrained class decreased both hard and soft neural network classification accuracy [4]. Therefore, the effect of untrained classes on SVDD and SVM classifications was also tested and compared.

**Figure 3:**
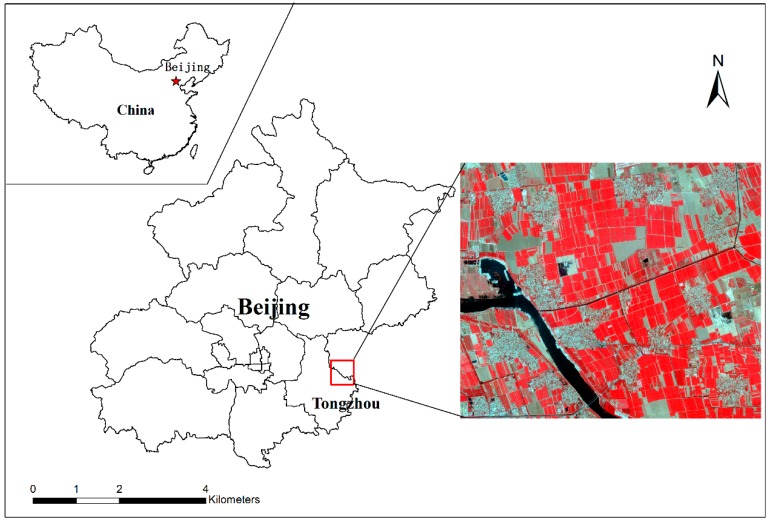
Study area and Quickbird (QB) imagery (bands combination = 4, 3, and 2).

A sub-region of Tongzhou, a district of southeast Beijing in China, was selected as study area, which covers an area of approximately 40 km^2^ (39°1′ N–39°4′ N, 116°2′ E–116°8′ E) (Figure 3). The topography is flat, and the fragmented agriculture land is a typical landscape in this area. The agriculture land is dominated by winter wheat. The other three primary land covers in this area, including trees, bare land and water, are mosaicked with the cultivated land.

High-quality multi-spectral Quickbird (QB) imagery at 2.4-m spatial resolution, with four bands (blue: 450–520 nm; green: 520–600 nm; red: 630–690 nm; NIR: 760–900 nm), acquired on 2 May 2006 under cloudless conditions, was used for this study. The pre-processing, atmospheric correction, was not necessary for the image due to the assumption that the atmosphere condition is uniform for one scene image which has little influence on classification conduction [29]. The geometric correction was applied for QB image to co-register with the field survey plot and projected to UTM (Universal Transverse Mercator) with the WGS-84 (World Geodetic System) coordinate system. To assess classification results, the actual land cover distribution was visually digitized directly from the original QB imagery as 2.4-m resolution of QB which was fine enough to support the accurate land cover extraction.

### 3.1. Comparison between WVS-SVDD, Conventional SVDD and SVM

In this section, we compared the WVS-SVDD with conventional SVDD approaches, and a binary SVM classification for mapping wheat and bare land, respectively. The comparison between WVS-SVDD and conventional SVDD was to observe the effect of window-based validation set on the classification performance. Besides, the SVM classification is considered as a basic benchmark for SVDD performance assessment, a common way for one-classifier accuracy analysis [15].

For conventional and WVS-SVDD classifications, a total of 120 training pixels, including 60 mixed derived along the target class boundary and 60 corner pixels extracted from the vertex of the MNF (minimum noise fraction) scatterplot, were chosen from each target class. Then, a validation set was acquired for each method. For the WVS-SVDD, a window with a size of 3 × 3 was fitted to each training pixel, the neighboring pixels of training samples within this window were collected to comprise validation set. The validation set was divided into target and outlier classes through visual interpretation. For conventional SVDD, the validation sets were randomly chosen through visual interpretation to collect target and outlier pixels. The numbers of target and outlier pixels in conventional validation sets were as same as those in the WVS-SVDD, and thus the accuracy discrepancy between WVS-SVDD and conventional SVDD could be illustrated from the spectral difference of the two validation sets. Optimal parameters were then determined using SA algorithm for each classification, shown in Table 1. SVDD-based land cover classifications were implemented using training sets and corresponding optimal parameters. Furthermore, a binary radial basis function (RBF)-based SVM classification using the optimal parameters (*C* and *s*) was implemented for mapping wheat [15]. In this approach, 120 pixels from the target class and another 120 pixels from the remaining land-cover classes were selected to comprise the training set. The results were finally assessed by confusion matrix measurement using the digitized wheat and bare land maps as test set, from which statistical metrics such as producer’s accuracy (PA), user’s accuracy (UA), overall accuracy (OA), and Kappa coefficient could be calculated [30].

**Figure 4:**
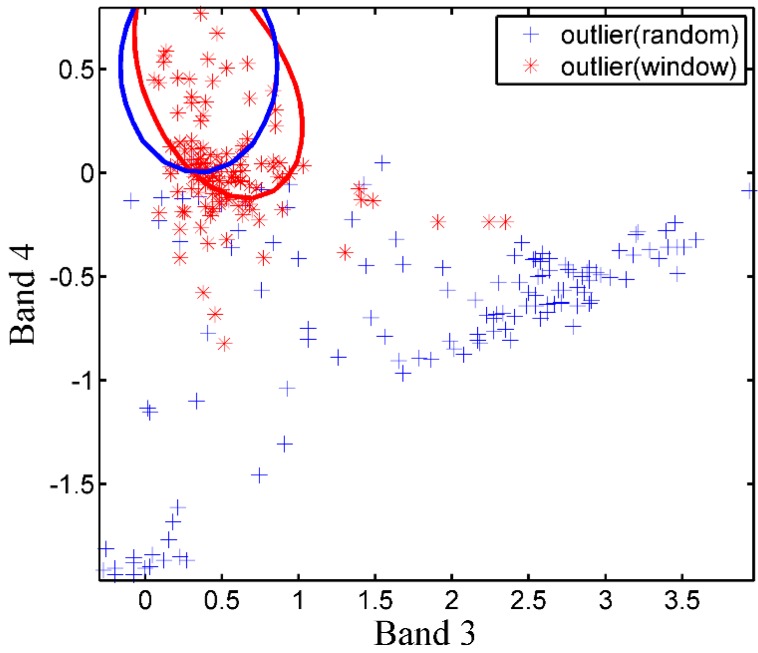
Hypersphere fitted by different validation sets. Red stars represent the validation data set derived from window-based method, and blue plus represents that from randomly selected method. Red ellipse represents hypersphere constructed by window-based test set while blue one by randomly selected test set.

In order to visualize the effect of window-based validation set on SVDD classification performance, the distribution of target pixels and outlier pixels in both validation sets for wheat mapping and its corresponding hyperspheres was given in Figure 4. For the traditional validation set, the randomly selected outlier pixels laid far away from the target class, thus an expansible hypersphere (*s* = 17.81) was achieved. In this case, those outliers that lay close to training samples were apt to be falsely accepted by the over-large hypersphere. For the window-based validation set, a compact hypersphere was constructed, mainly due to restriction by outlier pixels which were located neighboring to the target class.

**Figure 5:**
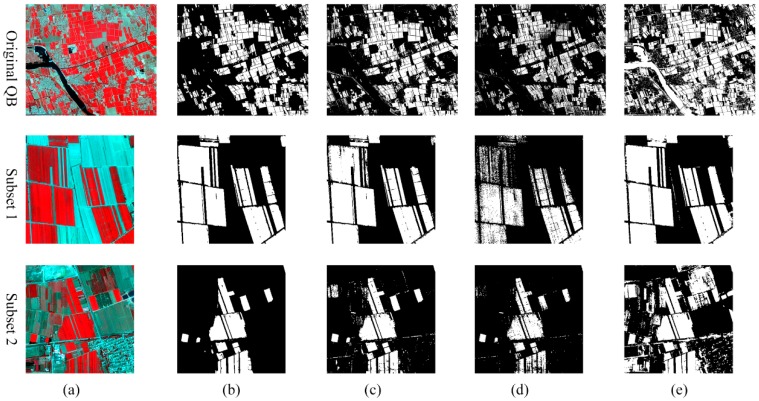
Comparison wheat classification results of different methods (**a**) Original remote sensing image; (**b**) reference result as actual data; (**c**) support vector machine (SVM) classification result; (**d**) WVS-SVDD classification result; (**e**) traditional SVDD method.

**Figure 6:**
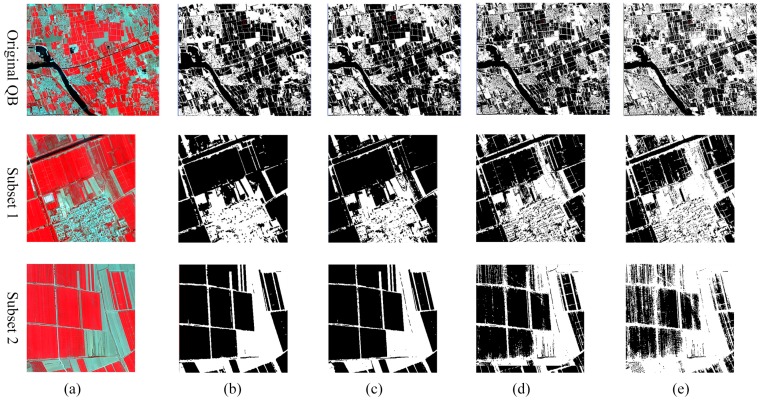
Comparison bare land classification results of different methods (**a**) Original remote sensing image; (**b**) reference result as actual data; (**c**) SVM classification result; (**d**) WVS-SVDD classification result; (**e**) traditional SVDD method.

SVDD and SVM classifications are given in Figure 5 and Figure 6 and the accuracy assessment was given in Table 2. For wheat, the binary SVM yielded an accurate performance with a satisfying overall accuracy of 94.37%. For the conventional SVDD-based wheat mapping, the omission error was only 3.94% because the constructed large hypersphere allowed accepting as many wheat pixels as possible. Unfortunately, the low omission error was achieved at the cost of a large commission error of 36.57%, causing a low overall accuracy under 80%. Comparing to the conventional method, the WVS-SVDD wheat classification yielded a more accurate classification result with an overall accuracy of 89.25%, at the same level with that derived from SVM classification. Less than 3% outliers were falsely accepted, highlighting the potential of outliers acquired from the window-based method for rejecting other classes. Classifications for bare land showed similar results. SVM classification performed best with an overall accuracy of 91.96% and WVS-SVDD yielded better accuracies than traditional method. Therefore, our proposed method could improve SVDD classification accuracy with more efficient validation sets. However, the omission errors of WVS-SVDD were relatively high because the constructed hypersphere was relatively small to completely enclose heterogeneous wheat spectral feature under 2.4-m resolution.

### 3.2. Sensitivity to Window Size and Pixels’ Spatial Scale

From above analysis, the WVS-SVDD-based hypersphere has shown excellent ability in rejecting outliers, however, the target class was underestimated at the window size of 3 × 3 because the constructed hypersphere covered a small space of target spectra. Furthermore, although the higher pixel resolution leads to a better delineation of land cover and reduces mixed pixels, land-cover classification maps using high-resolution images generally suffer noise problems due to high heterogeneity of spectral information at finer pixel size. Better classification accuracy may be achieved at a medium pixel size scale [31]. Thus, in this part, we evaluate the sensitiveness of the proposed method with respect to window size and pixels’ spatial scale.

The experiment setup is started by upscaling the QB image into low-resolution images, resulting in the land cover spectral features being more homogeneous. The original QB imagery was sub-resampled to 5-, 10-, 15-, and 20-m resolution according to the aggregated operation that aggregated the original image pixels’ spectrum [32]. Then, training sets were selected for each image, followed by validation set acquisition with different window sizes. Thus, in this paper, a variety of window sizes (5 × 5, 7 × 7, 9 × 9 and 11 × 11) were used to acquire more wheat pixels at the validation stage, which could increase the spectral variability of target pixels. WVS-SVDD classifications were carried out for the up-scaled data using the optimal parameters derived from SA algorithm under different spatial scales. To evaluate classification results generated from individual scales, the aggregated fraction map derived from the digitized wheat maps as the test set was used. These aggregated fraction maps were classified into binary wheat distribution maps using a majority rule in which pixels having faction higher than 0.5 were defined as “target class” and the remaining pixels as “outlier class” [33].

**Figure 7:**
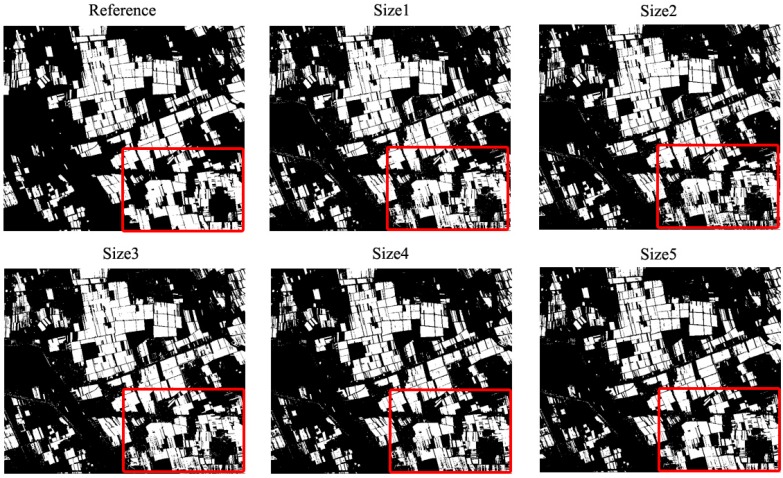
Comparison of SVDD classification results for wheat over different window sizes using simulated 10-m resolution image. The ground data are also included as reference.

Figure 7 and Table 3 showed WVS-SVDD classification results and accuracy assessment results using wheat reference maps, respectively. For spatial resolution at 2.4-m, the producer’s accuracy increased nearly 10% with the validation set search window size ranging from 3 to 7, meanwhile all user’s accuracies were higher than 95%. The overall accuracy also increased from 89.5% to 92.34%. The classification accuracy became stable after 7 × 7 scale, suggesting that a window size at 7 × 7 scale may be most suitable for choosing the validation data. The wheat spectra became more homogeneous and uniform, as the spatial resolution growing coarser, and target pixels derived in the window-based validation set with a size of 3 × 3 was generally enough to represent spectral characteristic of target class. As a result, more wheat pixels were correctly classified accompanying with producer’s accuracy increase. For each scenario, overall accuracies of WVS-SVDD classifications were greater than 88%, indicating that the proposed window-based validation set could help SVDD determine the optimal parameters, *C* and *s*, at an individual spatial scale.

### 3.3. The Effect of Untrained Classes on the Classification Accuracy

In this section, we chose a 10-m resolution image aggregated from original QB as an example and analyzed the performance of WVS-SVDD and SVM classification with untrained class. The training set for WVS-SVDD was same as in the above experiment, while bare land or trees were excluded in the validation set. Then, SVDD classifications were undertaken using optimal parameters determined by SA. There were six land cover combinations for the non-wheat training samples; detailed combinations are listed in Table 3. For SVM, 120 wheat training and 120 non-wheat training samples consisting of different non-wheat land covers combinations were chosen. SVDD and SVM classifications were then assessed using digitized wheat map.

**Figure 8:**
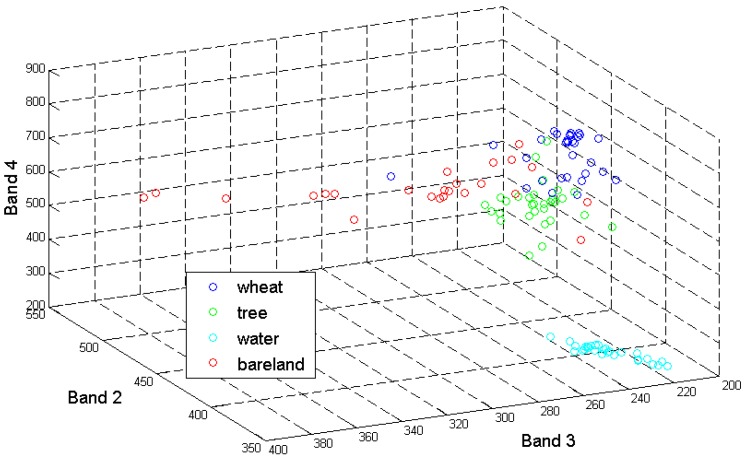
Three-dimensional scatterplot of land-cover types in the study area.

Table 4 showed accuracy of SVDD and SVM classification to map wheat. Under the condition of the use of training set with all defined classes, both SVDD and SVM achieved good performance with overall accuracy above 95%. However, the outlier class had obvious impacts, following reduction of classes in the non-wheat samples, on SVM classification accuracy. From the three-dimensional view of the scatterplot of land-cover types in Figure 8, wheat lies between bare land and trees, thus these two classes are necessary to construct an optimal hyperplane for separating wheat from other non-wheat land-cover types based on the principle of SVM. When trees or bare land were removed from the training set, classification accuracy decreased to some extent. In particular, the binary SVM derived the poorest classification with an overall accuracy of 76.28%, while neither trees nor bare land were included in the training set. The user’s accuracy was only 59.63%, indicating that the pixels representing areas of bare land and trees were commissioned into wheat. On the other hand, water is located far away from wheat, having little influences on the hyperplane. Therefore, excluding water in the training set had little impact on classification; overall accuracy was 96.01%, which was still at the same level with that derived by exhaustive training set. These results suggest that SVM is sensitive to the untrained class and an exhaustive training set is necessary to ensure an accurate classification. However, how to achieve the exhaustively defined training set is a big challenge. Instead, the WVS-SVDD still achieved good performance with high overall accuracy, with little sensitivity to whether trees or bare land pixels were excluded from the validation set, and was able to reach the same level of the SVDD classification as the original validation set. This is because the hypersphere constructed using the optimal *C* and *s* with support of window-based validation set could create the enclosed space to determine the wheat, suffering little influence from untrained non-wheat classes. Thus, WVS-SVDD is much less sensitive to the untrained classes than SVM, highlighting the value of SVDD for mapping one specific class which could significantly reduce the number of training pixels compared to multi-class classification where an exhaustive training set is required to keep classification accuracy. Compared to SVM, WVS-SVDD only requires few training and validation sets to ensure the target class classification performance.

## 4. Discussion

One-class classification using remote sensing is attracting more attention when users focus on one specific class. However, determining appropriate parameters to derive accurate land-cover maps has never been easy. This study proposed a window-based method to acquire informative outliers in the validation set which put more constraints on hypersphere compared to traditional randomly selected validation set. Figure 4 showed that outliers from WVS-SVDD provide effective outliers which were located tightly around the target training samples, resulting in an efficient hypersphere to reject non-target land-cover classes. Thus, the proposed method was able to significantly improve the overall accuracies for different land covers, which were higher than those derived from the traditional SVDD method, as shown in the Figure 5 and Figure 6.

Results from Table 3 exhibited that this algorithm proved to be robust for many spatial-resolution images. When image resolution gets coarser, land-cover spectra becomes more homogeneous and classification accuracies increased. This indicated that the proposed method can be applied to local-scale specific species mapping using high-resolution images and global-scale forest mapping using medium-resolution data.

Given results from Table 4, WVS-SVDD is less sensitive to the omission of any class in the validation set compared to multi-class SVM method. The reason is that SVDD does not need too many outlier classes to construct the hypersphere, whereas SVM needs to create hyperplanes between the target class and each outlier class. With this information, the training sample collection work can be reduced to a small percentage, which could enhance the classification efficiency.

However, there are still some limitations in the proposed method. For high-resolution image classification, more pixels should be included in the validation set due to higher spectral variation of land-cover classes, which induce heavier sample collection work. Also, this method has not been tested in urban environments which have a very complex mixture of different land-covers including vegetation, impervious surface and soil in the middle resolution images, such as Landsat 8 images. Moreover, buildings constructed with different materials and colors increase this complexity in the high-resolution images, such as UAV (Unmanned aerial vehicle) images. Thus, urban class is much more complicated than any other class presented in this study and the applicability of WVS-SVDD in urban environment will be explored in the future work.

## 5. Conclusions

This paper proposed a WVS-SVDD method that integrated a window-based validation set and an SA-based optimal *C* and *s* algorithm to map specific land cover. The results indicated that the proposed method performed much better than the conventional SVDD classification for one-class classification. For the wheat class, the overall accuracy of WVS-SVDD based classification was 89.25%, which was a little less than that derived from the SVM classification. Comparison between classifications for bare land showed similar results.

However, the underestimation of the target class indicated that the number of target pixels in the validation set is not enough at 3×3 window scale. Then, larger window sizes were adopted to select more wheat pixels to describe the wheat spectral responses. The study has shown that classification accuracy increased, accompanying with window size increment to some extent. Moreover, the proposed method was tested over various spatial patterns, varying from 2.4-m to 20-m resolution. Producer’s accuracy increased from 71.12% to 94.71%. Under such improvements, the commission error was keeping low, which was benefiting from the informative outliers. Therefore, the results highlighted the suitability of the proposed method over different spatial patterns.

The efficiency of untrained classes on the one- and multi-class classification was also analyzed in this paper. The SVM and WVS-SVDD classifications were undertaken with exhaustive or non-exhaustive training sets. For SVM classifications, exclusion of bare land or tree classes in the training set would decrease accuracy to some extent whereas excluding water had little influence on the classification. For WVS-SVDD, it still yielded good performance with high overall accuracy when trees or bare land pixels were excluded in the validation set, and was at the same level as that supported by the exhaustive validation set. These results suggested that SVDD is considerably less sensitive to the effect of untrained classes, thus SVDD is suitable for specific land-cover mapping with the limited and small validation set, compared to multi-class classification which requires exhaustive training set to ensure classification accuracy.

## Figures and Tables

**Table 1:** Numbers of target and outlier pixels in the validation sets for traditional and window-based validation set for support vector data description (WVS–SVDD) wheat and bare land classifications. The optimal parameters for each classification are also listed. *C*: tradeoff coefficient; *s*: kernel width.

Target Class	Classification Methods	# of Pixels in Validation Sets		Optimal Parameters	
		Target	Outlier	*C*	*s*
wheat	WVS-SVDD	470	128	0.02	1.01
	traditional SVDD			0.05	17.81
bare land	WVS-SVDD	449	68	0.08	9.98
	traditional SVDD			0.02	18.37

**Table 2:** Classification accuracy for the proposed, traditional and SVM methods. PA: producer’s accuracy; UA: user’s accuracy; OA: overall accuracy.

Target Class	Classification Methods	Classification Accuracy (%)		
		PA	UA	OA
wheat	WVS-SVDD	71.12	97.36	89.25
	Traditional Method	95.33	64.84	80.33
	SVM	94.57	90.37	94.37
bare land	WVS-SVDD	82.49	81.89	83.65
	Traditional Method	88.54	71.24	78.41
	SVM	90.85	91.51	91.96

**Table 3:** Accuracy assessment for each classification at different spatial patterns and window sizes.

Spatial Resolution and Window Size	# of Pixels in Validation Set		Optimal Parameters		Classification Accuracy (%)		
	Target	Outlier	*C*	*s*	PA	UA	OA
2.4 m							
3 ×3	470	128	0.02	1.01	71.12	97.36	89.25
5 × 5	1056	367	0.02	1.02	79.75	96.22	91.84
7 × 7	1687	702	0.05	3.76	80.49	97.05	92.34
9 × 9	2307	1104	0.02	1.40	81.18	95.62	92.13
11 × 11	2926	1580	0.01	1.03	80.11	96.11	91.93
5 m							
3 × 3	338	123	0.11	1.12	81.07	86.27	88.89
5 × 5	697	383	0.08	9.76	85.50	86.31	90.20
7 × 7	1090	748	0.21	7.37	81.91	93.66	91.75
9 × 9	1542	1202	0.21	1.94	82.32	93.42	91.80
11 × 11	2026	1744	0.09	4.37	85.10	86.73	90.25
10 m							
3 × 3	385	136	0.02	1.40	93.53	92.32	95.01
5 × 5	822	317	0.04	1.57	91.13	93.87	94.81
7 × 7	1302	554	0.02	1.51	92.93	92.53	94.90
9 × 9	1818	841	0.11	1.68	89.44	97.12	95.37
11 × 11	2358	1163	0.14	7.65	90.57	96.72	95.62
15 m							
3 × 3	464	144	0.01	1.04	91.92	93.10	94.75
5 × 5	1013	363	0.03	1.06	90.68	95.09	95.07
7 × 7	1657	905	0.02	1.08	92.07	93.48	94.95
9 × 9	2375	1480	0.02	1.11	91.63	94.39	95.14
11 × 11	3179	2176	0.03	1.09	90.87	95.13	95.15
20 m							
3 × 3	538	106	0.03	1.52	93.30	93.40	95.29
5 × 5	1198	302	0.03	1.47	93.39	93.41	95.33
7 × 7	1926	567	0.01	1.19	94.71	92.32	95.33
9 × 9	2694	913	0.03	1.51	93.33	93.41	95.30
11 × 11	3461	1329	0.02	1.21	93.88	93.87	95.66

**Table 4:** Classification accuracy for SVDD and SVM classifications using an exhaustive and non-exhaustive training data set.

Method	Untrained Class	Classification Accuracy (%)		
		PA	UA	OA
WVS-SVDD	None	93.53	92.32	95.01
	Bare land	94.40	85.93	92.63
	Trees	93.06	93.21	95.19
SVM	None	95.76	92.77	95.91
	Bare land	96.33	86.09	93.26
	Trees	98.81	81.51	91.73
	Water	95.17	93.55	96.01
	Trees and water	98.29	83.46	92.58
	Bare land and water	93.62	74.97	86.82
	Bare land and trees	99.98	59.63	76.28
